# Pegylated liposomal doxorubicin (Duomeisu^®^) monotherapy in patients with HER2-negative metastatic breast cancer heavily pretreated with anthracycline and taxanes: a single-arm, phase II study

**DOI:** 10.1007/s10549-023-06894-3

**Published:** 2023-03-06

**Authors:** Hanfang Jiang, Huiping Li, Guohong Song, Lijun Di, Bin Shao, Ying Yan, Xiaoran Liu, Yifei Chen, Ruyan Zhang, Ran Ran, Yaxin Liu, Xinyu Gui, Nan Wang, Huan Wang

**Affiliations:** Key Laboratory of Carcinogenesis and Translational Research (Ministry of Education), Department of Breast Oncology, Peking University Cancer Hospital & Institute, Beijing, China

**Keywords:** Pegylated liposomal doxorubicin, Metastatic breast cancer, HER2-negative, Efficacy, Safety

## Abstract

**Purpose:**

To evaluate the efficacy and safety of pegylated liposomal doxorubicin (PLD) in patients with human epidermal growth factor receptor 2 (HER2)-negative metastatic breast cancer (MBC) heavily pretreated with anthracycline and taxanes.

**Methods:**

In this single-arm, phase II study, patients with HER2-negative MBC previously treated with anthracycline and taxanes as second- to fifth chemotherapy received PLD (Duomeisu^®^, generic doxorubicin hydrochloride liposome) 40 mg/m^2^ every 4 weeks until disease progression, unacceptable toxicity, or completion of six cycles. Primary endpoint was progression-free survival (PFS). Secondary endpoints included overall survival (OS), objective response rate (ORR), disease control rate (DCR), clinical benefit rate (CBR), and safety.

**Results:**

Of 44 enrolled patients (median age, 53.5 years; range, 34–69), 41 and 36 were evaluable for safety and efficacy, respectively. In total, 59.1% (26/44) of patients had ≥ 3 metastatic sites, 86.4% (38/44) had visceral disease, and 63.6% (28/44) had liver metastases. Median PFS was 3.7 months (95% confidence interval [CI] 3.3–4.1) and median OS was 15.0 months (95% CI 12.1–17.9). ORR, DCR, and CBR were 16.7%, 63.9%, and 36.1%, respectively. The most common adverse events (AEs) were leukopenia (53.7%), fatigue (46.3%), and neutropenia (41.5%), with no grade 4/5 AEs. The most common grade 3 AEs were neutropenia (7.3%) and fatigue (4.9%). Patients experienced palmar-plantar-erythrodysesthesia (24.4%, 2.4% grade 3), stomatitis (19.5%, 7.3% grade 2), and alopecia (7.3%). One patient displayed a left ventricular ejection fraction decline of 11.4% from baseline after five cycles of PLD therapy.

**Conclusion:**

PLD (Duomeisu^®^) 40 mg/m^2^ every 4 weeks was effective and well-tolerated in patients with HER2-negative MBC heavily pretreated with anthracycline and taxanes, revealing a potentially viable treatment option for this population.

**Trial registration** Chinese Clinical Trial Registry: ChiCTR1900022568.

**Electronic supplementary material:**

The online version of this article (10.1007/s10549-023-06894-3) contains supplementary material, which is available to authorized users.

## Introduction

Breast cancer has become the most commonly diagnosed cancer globally in 2020, surpassing lung cancer (11.4%), with an estimated 2,261,419 new cases (11.7%), and the fourth leading cause (6.9%) of cancer-related deaths worldwide, with an estimated 684,996 new deaths in 2020 [1]. Approximately 20–50% of patients with early breast cancer will eventually develop metastatic disease [2], and 5–10% of patients are initially diagnosed with metastatic disease [3]. Among patients with metastatic breast cancer (MBC), 62% are diagnosed with human epidermal growth factor receptor 2 (HER2)-negative disease [4]. Despite considerable advances in new treatments, MBC remains treatable but incurable, with the goal of treatment to prolong patients’ survival and improve their quality of life [5, 6].

During the past 70 years, the highest number of new drugs for breast cancer was approved [7]. Nevertheless, conventional anthracyclines (doxorubicin and epirubicin) and taxanes remain the cornerstones of breast cancer therapy regardless of the molecular subtype, whether in the neoadjuvant/adjuvant setting or metastatic setting[6, 8–12]. However, anthracycline- and taxane-based regimens are strongly recommended as preferred chemotherapy options in the neoadjuvant/adjuvant setting [10, 13–15]. In the metastatic setting, taxane-based chemotherapy is commonly used as first-line therapy. Anthracycline reuse in the metastatic setting is limited by toxicity, especially cumulative, dose-related, irreversible cardiotoxicity [16–18], although rechallenge with anthracycline previously used in the neoadjuvant/adjuvant setting, is considered a reasonable option under certain conditions [11, 12].

Pegylated liposomal doxorubicin (PLD), developed in an attempt to reduce the toxicity profile of conventional doxorubicin while preserving its anti-tumor efficacy, became the first United States Food and Drug Administration -approved cancer nanomedicine in 1994, and it is currently indicated for advanced ovarian cancer, acquired immune deficiency syndrome (AIDS)-related Kaposi’s sarcoma, and multiple myeloma in the United States [19]. In Europe, PLD is also indicated for MBC [19]. PLD is a unique formulation of doxorubicin encapsulated by polyethylene glycol-coated liposomes, with a longer circulation time, higher selective accumulation in tumors, lower cardiac toxicity, less alopecia, myelosuppression, nausea, and vomiting in comparison with conventional doxorubicin[19–23]. Various clinical studies confirmed that PLD monotherapy or combination therapy is an effective first-line regimen with a good safety profile in both HER2-negative and HER2-positive MBC [22–30]. Moreover, single-agent PLD or PLD-based therapy demonstrated efficacy and safety as a selectable salvage regimen in patients with conventional anthracycline- and/or taxane-pretreated MBC [31–37]. However, no clinical studies have evaluated the efficacy and safety of single-agent PLD in patients with HER2-negative MBC previously treated with conventional anthracycline and taxanes.

Additionally, the recommended dose of PLD is 50 mg/m^2^ day 1 every 4 weeks as one of the preferred regimens for stage IV/recurrent metastatic HER2-negative breast cancer by the National Comprehensive Cancer Network clinical guidelines for breast cancer [38]. However, PLD 50 mg/m^2^ every 4 weeks is associated with higher rates of palmar-plantar-erythrodysesthesia (PPE; all grades: 48% vs. 2%; ≥ grade 3: 17% vs. 0%) and stomatitis (all grades: 22% vs. 15%; ≥ grade 3: 5% vs. 2%) than the equivalent conventional doxorubicin 60 mg/m^2^ every 3 weeks [22]. These toxicities are especially less acceptable for patients with heavily pretreated MBC. A previous phase II study in Germany demonstrated PLD 40 mg/m^2^ every 4 weeks as a salvage regimen has similar efficacy and lower rates of PPE and stomatitis than PLD 50 mg/m^2^ every 4 weeks in patients with pretreated MBC[39].

Duomeisu^®^ is a generic doxorubicin hydrochloride liposomal formulation manufactured by Shijiazhuang Pharmaceutical Group Ouyi Pharmaceutical Co. Ltd. (Shijiazhuang, China), approved for AIDS-related Kaposi’s sarcoma in 2012 by National Medical Products Administration in China.

We conducted a prospective, single-center, open-label, phase II study to evaluate the efficacy and safety of Duomeisu^®^ 40 mg/m^2^ every 4 weeks in patients with HER2-negative MBC previously treated with conventional anthracycline and taxane in China.

## Patients and methods

### Patients

Between July 2017 and July 2021, female patients with histologically confirmed HER2-negative MBC previously treated with conventional anthracyclines and taxanes were enrolled in this study. HER2 negativity was defined as a 0 or 1 + on immunohistochemistry and/or negativity by fluorescence in situ hybridization in a primary or metastatic tumor sample. Other eligibility criteria included age ≥ 18 and ≤ 70 years; at least one but no more than four lines of prior systemic chemotherapy for metastatic disease; at least one measurable lesion according to Response Evaluation Criteria for Solid Tumors (RECIST) version 1.1[40]; Eastern Cooperative Oncology Group performance status of 0–2; life expectancy ≥ 3 months; left ventricular ejection fraction (LVEF) ≥ 50% by echocardiography; adequate bone marrow (absolute neutrophil count ≥ 1.5 × 10^9^/L, platelet count ≥ 100 × 10^9^/L, hemoglobin ≥ 90 g/L), hepatic (total serum bilirubin ≤ 1.5 × upper limit of normal (ULN), aspartate transaminase and/or alanine transaminase ≤ 3.0 × ULN [≤ 5.0 × ULN if liver metastasis]) and renal function (serum creatinine ≤ 1.5 × ULN); and negativity on a serum or urine pregnancy test and willingness to use highly effective methods of contraception to prevent pregnancy for potential child-bearing patients during and 3 months following treatment cessation. Bisphosphonate therapy was permitted when entering the study.

Exclusion criteria were as follows: congestive heart failure (New York Heart Association class II or higher); untreated or uncontrolled brain metastases; severe systemic infection; allergy to PLD or other related supplementary materials; prior anthracycline exceeding a total cumulative dose of 300 mg/m^2^ doxorubicin or 550 mg/m^2^ epirubicin or equivalent, or heart disease due to the use of anthracyclines; history of other malignancies (excluding cured cervical cancer or skin basal cell carcinoma) within the last 5 years; receipt of other anti-tumor therapies or other experimental drugs within 28 days.

This study was approved by the Ethics Committee of Peking University Cancer Hospital (Approval ID: 2017YJZ08) in Beijing, China, and was registered in the Chinese Clinical Trial Registry (ChiCTR1900022568). This study was conducted according to the Declaration of Helsinki and Good Clinical Practice Guidelines. All participants provided written informed consent.

### Study design and treatment

This was a prospective, single-center, open-label, single-arm, phase II study designed to evaluate the efficacy and safety of PLD 40 mg/m^2^ every 4 weeks monotherapy in patients with HER2-negative MBC pretreated with conventional anthracycline and taxanes. Patients received PLD (Duomeisu^®^) 40 mg/m^2^ diluted in 250 mL of 5% dextrose intravenous infusion for 1 h on day 1 of each 28-day cycle. The detailed dose adjustment schemes were presented in Supplemental Methods. Treatment was continually administered until disease progression, unacceptable toxicity, treatment delay of > 3 weeks owing to toxicity, completion of 6 cycles, or patient’s decision to withdraw from the study.

### Study objectives and assessment

The primary objective of the study was to evaluate progression-free survival (PFS) in the intent-to-treat (ITT) population. PFS was defined as the time from the first dose of PLD to disease progression per the RECIST 1.1 criteria, death from any cause without progression, or the last follow-up visit without progression. The secondary objectives included overall survival (OS), objective response rate (ORR), disease control rate (DCR), clinical benefit rate (CBR), and safety. OS was defined as the time from the first dose of PLD to death from any cause, and it was censored at the date of the last follow-up. ORR was defined as the percentage of patients with the best overall response of complete response (CR) or partial response (PR) per the RECIST 1.1 criteria (without confirmation), DCR was defined as the percentage of patients with CR, PR, or stable disease (SD), and CBR was defined as the percentage of CR, PR, or SD for at least 24 weeks. Tumor responses were accessed by computer tomography/magnetic resonance imaging every two cycles (every 8 weeks) during treatment and every 3 months after discontinuation until disease progression according to RECIST version 1.1[40], as assessed by the investigator. Echocardiography was performed to measure LVEF at baseline, 1 day before cycles 3 and 5, at the end of the treatment, and 3 and 6 months after completing study treatment. The safety assessment was based on the frequency and severity of adverse events (AEs) according to the National Cancer Institute Common Terminology Criteria for Adverse Events (CTCAE, version 4.0). AEs were recorded on the first day of every cycle and at the treatment discontinuation visit. Patients were followed up every 3 months until death from any cause.

### Statistics

The sample size was calculated using the one-sample log-rank test based on the primary endpoint of PFS. According to Al-Batran’s study [39], median PFS was 3.3 months in the control group. Assuming a Weibull distribution shape parameter of *k* = 1, target hazard ratio of 0.667, two-sided alpha level of 5%, and power of 80%, the planned enrollment and follow-up periods were both 12 months. Considering these parameters, a sample size of the least 43 patients was necessary. Therefore, we aimed to recruit 45 patients.

Patients who received at least two doses of PLD and completed at least one efficacy evaluation were included in the efficacy analysis. Safety was analyzed by descriptive statistics in all patients who received at least one dose of PLD and underwent at least one safety evaluation. PFS and OS analyses were estimated using the Kaplan–Meier method, and 95% confidence intervals (CIs) were also calculated. ORR, DCR, and CBR were summarized with their 95% CIs. Descriptive quantitative data were expressed as median and range according to the data distribution, and qualitative data were expressed as counts and percentages. Two-sided *p* values were reported and *p* values less than 0.05 were considered significant. All analyses were performed using SPSS version 20.0 (SPSS, Inc., Chicago, IL, USA).

## Results

### Patient characteristics

**Fig. 1:**
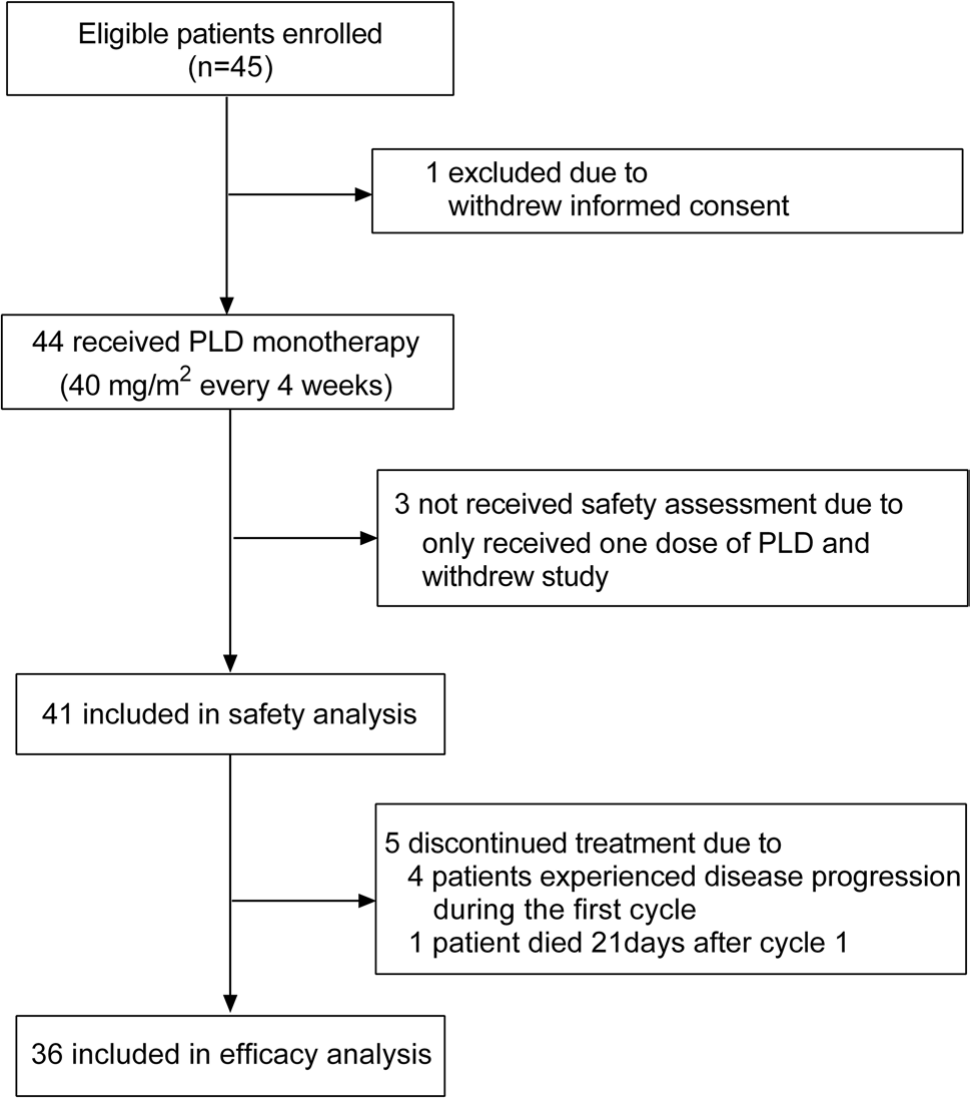
The CONSORT diagram

Between July 2017 and July 2021, a total of 45 patients were recruited for this study. One patient withdrew from this study before receiving treatment. Three patients received only one dose of PLD and withdrew from this study without a safety assessment. Therefore, 41 patients were included in the safety analysis. One patient died 21 days after the first dose because of disease progression, and four patients experienced disease progression during the first cycle. Finally, 36 patients who completed at least two cycles of PLD were included in the efficacy analyses. At the data cut-off date of November 2021, the median follow-up time was 13.2 months (range 2.4–48.0 months). Figure 1 depicts the CONSORT diagram of this study.

The demographics and baseline characteristics of the patients are presented in Table 1 (*N* = 44). The median age was 53.5 years (range, 34–69 years). 11 (25.0%) patients were premenopausal, and 32 (75.0%) were postmenopausal. Notably, 25.0% (11/44) of patients had hormone receptor (HR)-negative tumors. 59.1% (26/44) of patients had ≥ 3 metastatic sites, 86.4% (38/44) had visceral disease, and 63.6% (28/44) had liver metastases. All patients had received conventional anthracycline and taxane previously in the neo/adjuvant setting (37/44, 84.1%), metastatic setting (6/44, 13.6%), or both settings (1/44, 2.3%). Seventeen patients (38.7%) received ≥ 3 prior systemic chemotherapies for metastatic disease, and the median number of prior chemotherapy regimens in the metastatic setting was two (range 1–4). The median anthracycline-free interval (time from the last dose of previous anthracycline to enrollment in this trial) and median PFS of the last therapy were 59.5 months (range 10.0–212.0) and 3.0 months (range 1.0–18.0), respectively.

**Table 1:** Patient demographics and baseline characteristics (*n* = 44)

Patient characteristics	*n* = 44
Age (years, median/range)	53.5 (34–69)
Gender (*n*, %)	
Female	44 (100)
Menopause status (*n*, %)	
Premenopausal	11 (25.0)
Postmenopausal	33 (75.0)
ECOG performance status at inclusion (*n*, %)	
0	41 (93.2)
1	3 (6.8)
Receptor status at diagnosis (*n*, %)	
Estrogen receptor-positive	30 (68.2)
Progesterone receptor-positive	31 (70.5)
Triple-negative^a^	11 (25.0)
Metastatic site (*n*, %)	
Bone	27 (61.4)
Lymph nodes	29 (65.9)
Soft tissues	13 (29.5)
Lung	20 (45.5)
Pleura	5 (11.4)
Liver	28 (63.6)
Brain	2 (4.5)
Site of disease (*n*, %)	
Visceral	38 (86.4)
Nonvisceral	6 (13.6)
Number of metastatic sites (*n*, %)	
1	6 (13.6)
2	12 (27.3)
≥ 3	26 (59.1)
Prior lines of chemotherapy for metastatic disease (median, range)	2 (1–4)
Number of prior metastatic chemotherapy regimens (*n*, %)	
1	14 (31.8)
2	13 (29.5)
≥ 3	17 (38.7)
Previous metastatic regimens (*n*, %)	
Epirubicin	6 (15.9)
Pirarubicin	1 (2.3)
Docetaxel	23 (52.3)
Paclitaxel	16 (36.4)
Liposomal paclitaxel	5 (11.4)
Nab-paclitaxel	7 (15.9)
Capecitabine	31 (70.5)
Gemcitabine	20 (45.5)
Vinorelbine	16 (36.4)
Platinum	11 (25.0)
Etoposide	3 (6.8)
Hormone therapy	27 (61.4)
Targeted therapy	15 (34.1)
Previous metastatic chemotherapy (*n*, %)	44 (100)
Monotherapy	11 (25.0)
Combined therapy	33 (75.0)
Prior anthracycline and taxane-based therapy	44 (100)
Previous chemotherapy in neo/adjuvant setting (*n*, %)	
Anthracycline-based	13 (29.5)
Anthracycline and taxane-based	25 (56.8)
Previous chemotherapy in the metastatic setting (*n*, %)	
Taxane-based	34 (77.3)
Anthracycline and taxane-based	7 (15.9)
Setting of prior conventional anthracycline	
Neo/adjuvant	37 (84.1)
Metastatic	6 (13.6)
Both	1 (2.3)
Prior conventional anthracycline	
Doxorubicin	7 (15.9)
Epirubicin	30 (68.2)
Pirarubicin	7 (15.9)
Cumulative dose of prior conventional anthracycline (mg/m^2^, median/range)	
Doxorubicin	195 (155–294)
Epirubicin	380 (225–530)
Pirarubicin	220 (170–342)
Anthracycline-free interval	
0–12 months	2 (4.5)
> 12 months	42 (95.5)
Interval from the last dose of previous anthracycline to the time of enrollment of this trial (months, median/range)	59.5 (10.0–212.0)
Baseline LVEF (mean, range)	66 (57–73)
PFS of last therapy (months, median/range)	3.0 (1.0–18.0)

*ECOG* Eastern Cooperative Oncology Group, *LVEF* left ventricular ejection fraction, *PFS* progression-free survival.

^a^Estrogen receptor, progesterone receptor, and human epidermal growth factor receptor 2 negative.

### Efficacy

Thirty-six patients were assessable for clinical response. Ten patients (27.8%) completed the planned six cycles, and 26 patients (72.2%) discontinued treatment earlier than planned: 22 patients (61.1%) because of disease progression, 2 patients (5.6%) because of AEs, and 2 patients due to patient choices (Fig. 1). Among 10 patients who completed the planned six cycles of PLD, six discontinued for follow-up, and four withdrew from this trial, including two continued to receive PLD until disease progression (four cycles, *n* = 1; two cycles, *n* = 1), one who experienced disease progression after receiving two months of toremifene maintenance therapy, and one patient underwent modified radical mastectomy for the primary breast lesion after CR of distant metastases following six cycles of PLD.

**Fig. 2:**
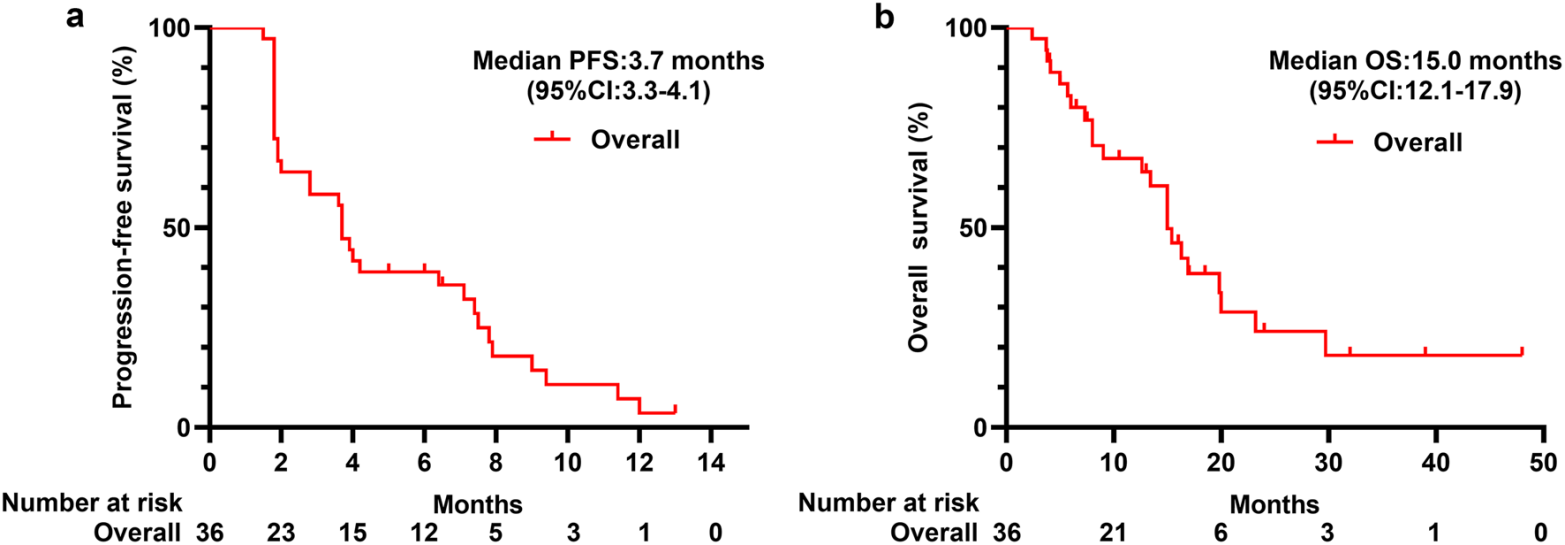
Kaplan–Meier plots for **a** progression-free survival in all patients (*n* = 36), **b** overall survival in all patients (*n* = 36). *CI* confidence interval, *PFS* progression-free survival, *OS* overall survival

**Fig. 3:**
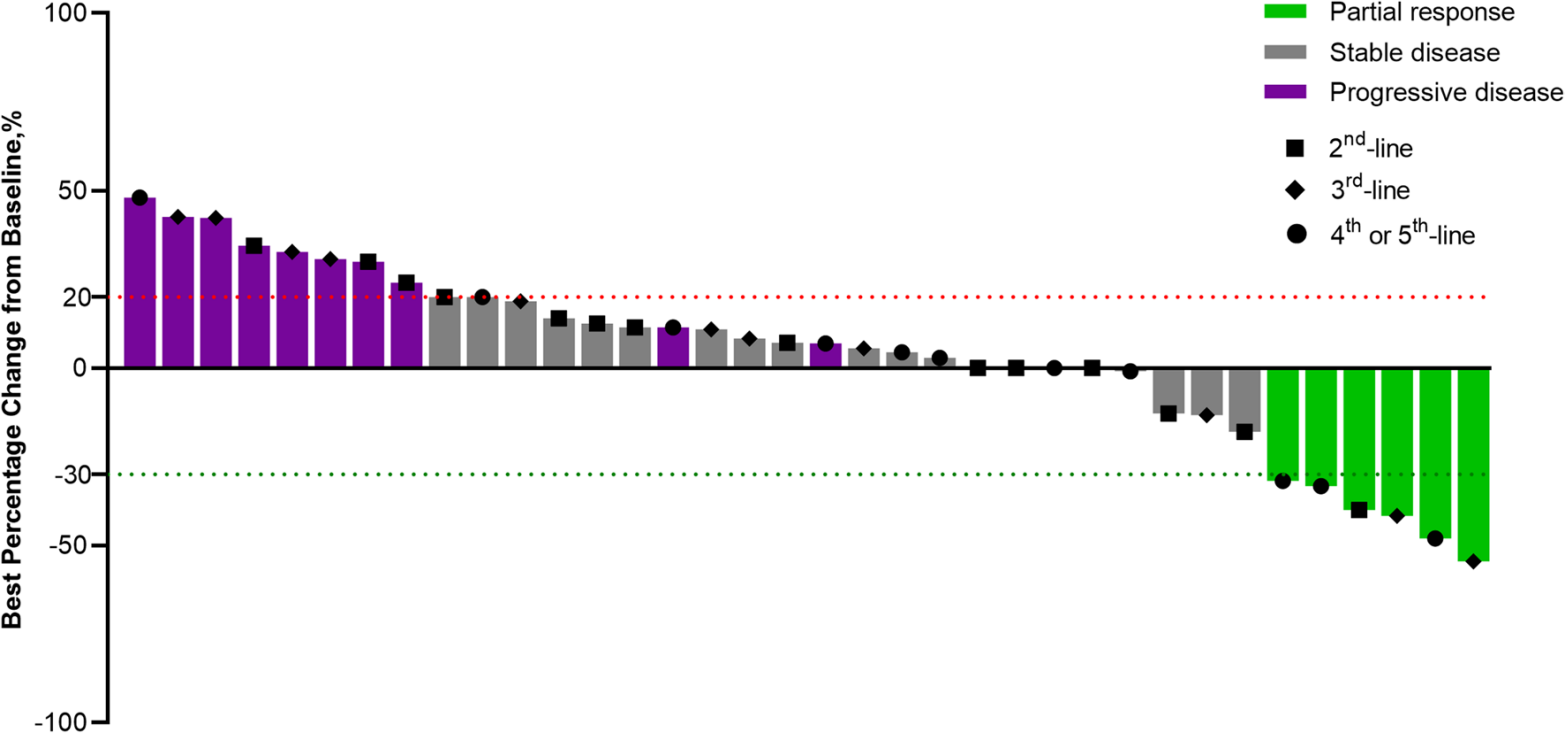
Waterfall plot of the maximum percent change in tumor size from baseline in each patient as per RECIST 1.1

Median PFS was 3.7 months (95% CI 3.3–4.1) (Fig. 2a), and median OS was 15.0 months (95% CI 12.1–17.9) (Fig. 2b), respectively. No patients had CR, whereas six patients (16.7%) had PR. ORR, DCR, and CBR were 16.7% (95% CI 6.4–32.8), 63.9% (95% CI 46.2–79.2), and 36.1% (95% CI 20.8–53.8), respectively. Figure 3 provides additional details regarding the depth of response.

**Fig. 4:**
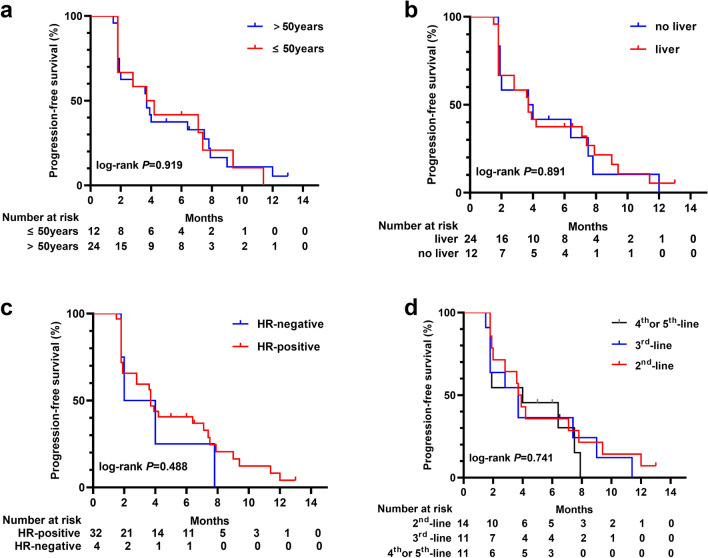
Kaplan–Meier plots for progression-free survival in subgroup based on the patients' demographics and baseline characteristics, including** a** age, **b** liver metastasis,** c** hormone receptor status, and** d** treatment line

**Fig. 5:**
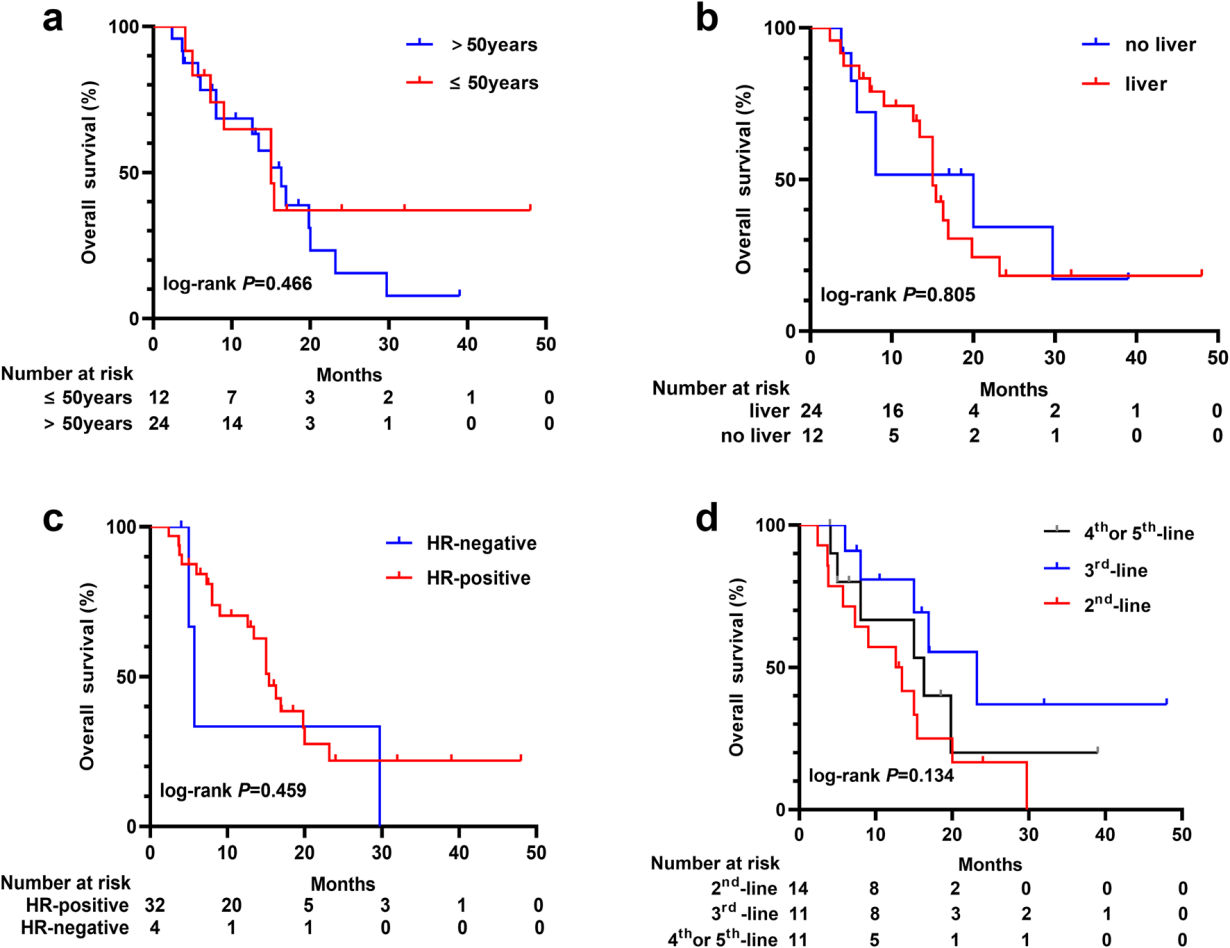
Kaplan–Meier plots for overall survival in subgroup based on the patients’ demographics and baseline characteristics, including **a** age, **b** liver metastasis, **c** hormone receptor status, and **d** treatment line

The subgroup analysis of PFS or OS according to age (≤ 50 years vs. > 50 years), liver metastasis, HR status, and the treatment line (second-line, third-line, or fourth/fifth-line was performed. There were no statistically significant differences in subgroup analysis for the PFS (Fig. 4) or OS (Fig. 5) (all *p* > 0.05). The median PFS times were 3.7, 3.7, and 4.0 months in the second, third, and fourth/fifth lines, respectively (Fig. 4d). The median OS in the second, third, and fourth/fifth lines were 12.6, 23.2, and 16.3 months, respectively (Fig. 5d). Efficacy results are summarized in Table 2.

**Table 2:** Summary of the efficacy data (n = 36)

	Total (*n* = 36)	2nd - line (*n* = 14)	3rd - line (*n* = 11)	≥ 4th - line (*n* = 11)
Tumor response (*n*, %)				
CR	0 (0)	0 (0)	0 (0)	0 (0)
PR	6 (16.7)	1 (7.1)	2 (18.2)	3 (27.3)
SD	17 (47.2)	9 (64.3)	5 (45.5)	3 (27.3)
PD	13 (36.1)	4 (28.6)	4 (36.4)	5 (45.5)
ORR (CR + PR)	6 (16.7)	1 (7.1)	2 (18.2)	3 (27.3)
DCR (CR + PR + SD)	23 (63.9)	10 (71.4)	7 (63.6)	6 (54.5)
CBR (CR + PR + SD ≥ 6 months)	13 (36.1)	5 (35.7)	4 (36.4)	4 (36.4)
PFS (months, median/95% CI)	3.7 (3.3–4.1)	3.7 (3.2–4.3)	3.7 (1.7–5.7)	4.0 (0.0–8.1)
OS (months, median/95% CI)	15.0 (12.1–17.9)	12.6 (5.2–20.0)	23.2 (10.5–35.9)	16.3 (5.6–27.0)

*CR* complete response, *PR* partial response, *SD* stable disease, *PD* progressive disease, *ORR* objective response rate, *DCR* disease control rate, *CBR* clinical benefit rate, *PFS* progression-free survival, *CI* confidence interval, *OS* overall survival

### Safety

Of 44 patients enrolled, 41 who received at least one dose of PLD were evaluable for safety. PLD was administered for a mean of 3.4 cycles (range, 1–6). The mean cumulative dose of PLD was 134 mg/m^2^ (range, 40–240). The median dose intensity of PLD was 10 mg/m^2^/week (range, 8.3–10). Dose reduction occurred in four patients (9.8%) because of grade 2/3 PPE (4.9%), grade 3 fatigue (4.9%), and grade 2 palpitation (2.4%). Dose delay occurred in two patients (4.9%) because of grade 3 neutropenia. Two patients (4.9%) discontinued treatment after the fifth cycle of PLD due to grade 3 fatigue (2.4%), and grade 2 dyspnea with an 11.4% LVEF decline compared with baseline (2.4%).

Forty (97.6%) patients reported AEs. The most common all-grade AEs included leukopenia (53.7%), fatigue (46.3%), neutropenia (41.5%), nausea (31.7%), and aspartate aminotransferase elevation (29.3%). Overall, treatment was well-tolerated with no grade 4/5 AEs. Nine patients (22.0%) experienced grade 3 AEs, including neutropenia (7.3%), fatigue (4.9%), PPE (2.4%), anemia (2.4%), vomiting (2.4%), and constipation (2.4%). Patients reported PPE (24.4%, 2.4% grade 3), stomatitis (19.5%, 7.3% grade 2), and alopecia (7.3%) during treatment. There was no evidence of marked LVEF decreases in 40 patients (97.6%) during treatment. Only one patient observed LVEF declined from 70% at baseline to 62% after cycle 5 of PLD (11.4% drop from baseline, grade 2). No other cardiac AEs were observed. AEs are listed in Table 3.

**Table 3:** Treatment-related adverse events (*n* = 41)

Events	All grades (*n*, %)	Grade 1 (*n*, %)	Grade 2 (*n*, %)	Grade 3 (*n*, %)	Grade 4 (*n*, %)
Hematological					
Leukopenia	22 (53.7)	14 (34.1)	8 (19.5)	0 (0)	0 (0)
Neutropenia	17 (41.5)	6 (14.6)	8 (19.5)	3 (7.3)	0 (0)
Anemia	9 (22.0)	7 (17.1)	1 (2.4)	1 (2.4)	0 (0)
Thrombocytopenia	1 (2.4)	1 (2.4)	0 (0)	0 (0)	0 (0)
Non-hematological					
Alopecia	3 (7.3)	3 (7.3)	0 (0)	0 (0)	0 (0)
Dyspepsia	5 (12.2)	2 (4.9)	2 (4.9)	1 (2.4)	0 (0)
Dysphagia	2 (4.9)	2 (4.9)	0 (0)	0 (0)	0 (0)
Weight loss	3 (7.3)	3 (7.3)	0 (0)	0 (0)	0 (0)
Abdominal distension	3 (7.3)	3 (7.3)	0 (0)	0 (0)	0 (0)
Nausea	13 (31.7)	13 (31.7)	0 (0)	0 (0)	0 (0)
Vomiting	8 (19.5)	6 (14.6)	1 (2.4)	1 (2.4)	0 (0)
Diarrhea	1 (2.4)	1 (2.4)	0 (0)	0 (0)	0 (0)
Constipation	6 (14.6)	4 (9.8)	1 (2.4)	1 (2.4)	0 (0)
Anorexia	10 (24.4)	8 (19.5)	2 (4.9)	0 (0)	0 (0)
Fatigue	19 (46.3)	14 (34.1)	3 (7.3)	2 (4.9)	0 (0)
Insomnia	10 (24.4)	10 (24.4)	0 (0)	0 (0)	0 (0)
Fever	2 (4.9)	2(4.9)	0 (0)	0 (0)	0 (0)
Stomatitis	8 (19.5)	5 (12.2)	3 (7.3)	0 (0)	0 (0)
Pigmentation of skin	8 (19.5)	8 (19.5)	0 (0)	0 (0)	0 (0)
ALT elevation	7 (17.1)	7 (17.1)	0 (0)	0 (0)	0 (0)
AST elevation	12 (29.3)	11 (26.8)	1(2.4)	0 (0)	0 (0)
Bilirubin increased	4 (9.8)	4 (9.8)	0 (0)	0 (0)	0 (0)
Hyperglycemia	2 (4.9)	2 (4.9)	0 (0)	0 (0)	0 (0)
Cholesterol high	4 (9.8)	4 (10.0)	0 (0)	0 (0)	0 (0)
Palpitation	2 (4.9)	1 (2.4)	1 (2.4)	0 (0)	0 (0)
Dyspnea	3 (7.5)	2 (4.9)	1 (2.4)	0 (0)	0 (0)
Neurosensory	9 (22.0)	9 (22.0)	0 (0)	0 (0)	0 (0)
Dysgeusia	5 (12.2)	4 (9.8)	1 (2.4)	0 (0)	0 (0)
Dry skin	8 (19.5)	8 (19.5)	0 (0)	0 (0)	0 (0)
Pruritus	4 (9.8)	4 (9.8)	0 (0)	0 (0)	0 (0)
Rash	2 (4.9)	2 (4.9)	0 (0)	0 (0)	0 (0)
Myalgia	10 (24.4)	9 (22.0)	1 (2.4)	0 (0)	0 (0)
Bone pain	2 (4.9)	1 (2.4)	1 (2.4)	0 (0)	0 (0)
Edema limbs	1 (2.4)	1 (2.4)	0 (0)	0 (0)	0 (0)
Pigmentation	8 (19.5)	8 (19.5)	0 (0)	0 (0)	0 (0)
PPE	10 (24.4)	8 (19.5)	1 (2.4)	1 (2.4)	0 (0)
LEVF decrease	1 (2.4)	0 (0)	1 (2.4)	0 (0)	0 (0)

*ALT* alanine aminotransferase, *AST* aspartate aminotransferase, *LEVF* left ventricular ejection fraction, *PPE* Palmar-plantar-erythrodysesthesia

## Discussion

This study first evaluated the efficacy and safety of Duomeisu^®^ monotherapy at 40 mg/m^2^ every 4 weeks in Chinese women with HER2-negative MBC heavily pretreated with conventional anthracyclines and taxanes. The results of this study revealed that median PFS was 3.7 months (95% CI 3.3–4.1) with an ORR of 16.7% and CBR of 36.1%, whereas median OS was 15.0 months (95% CI 12.1–17.9). Most AEs were mild or moderate, with no grade 4/5 AEs. Grade 3 AEs were infrequent, with 7.35% neutropenia, 4.9% fatigue, and 2.4% PPE.

An unmet need exists for patients with heavily pretreated HER2-negative MBC. After anthracycline and taxane failure, there is no optimal chemotherapy regimen established for such patients [41]. Single-agent PLD has been demonstrated to be effective and safe for the treatment of MBC [22, 31, 39, 42, 43]. For patients with pretreated MBC, a previous prospective phase 2 study of PLD 40 mg/m^2^ every 4 weeks as a second- or third-line treatment by Al-Batran et al. [39] recorded median PFS of 3.3 months (95% CI 2.8–5.4), and median OS of 10.7 months (95% CI 6.0 –18.3). In this trial, 71.7% of the patients had prior anthracycline exposure, and 54.3% had prior taxane exposure. Another multicenter phase II study of patients with MBC all previously treated with conventional anthracyclines and 68.4% with prior taxane exposure who received PLD 50 mg/m^2^ every 4 weeks showed that the PFS and OS were 3.6 months (95% CI 2.7–6.4) and 12.3 months (95% CI 7.7–16.3), respectively [33]. Similarly, in an earlier phase III study of PLD versus vinorelbine or mitomycin C plus vinblastine as a second-third-line treatment for patients with taxane-refractory MBC, 17% of whom were anthracycline-naïve, PLD 50 mg/m^2^ every 4 weeks achieved median PFS of 2.9 months and median OS of 11.0 months [31]. In our study, median PFS of 3.7 months (95% CI 3.3–4.1) and median OS of 15.0 months (95% C: 12.1–17.9) were numerically longer than those of the aforementioned trials. Of note, all patients had received prior conventional anthracycline and taxane in our study. Moreover, 86.4% of these patients had visceral disease, 63.6% liver metastases, and 38.7% received ≥ 3 prior systemic chemotherapies for metastatic disease. Additionally, the median PFS of the last therapy was only 3.0 months. Thus, the enrolled patients had a particularly poor prognosis in our study. The results in the present study suggested anthracycline- and taxane-resistant MBC is not cross-resistant to PLD, similar to the findings of a randomized phase III study of patients with taxane-refractory advanced breast cancer [31]. Furthermore, promising anti-tumor activity was also observed even in the fourth/ fifth-line group, with median PFS of 4.0 months and median OS of 16.3 months in the present study, indicating that PLD could be used in patients with heavily pretreated HER2-negative MBC. Overall, a reduction of the PLD dose to 40 mg/m^2^ every 4 weeks in our study was not associated with worse survival outcomes. Conversely, numerically longer OS was observed in this study. This discrepancy might be explained by patients with advanced breast cancer in earlier studies including the HER2-positive subtype and the likely superiority of Duomeisu^®^ to DOXIL^®^ (Ortho Biotech Products, L.P.) or Caelyx^®^ (Janssen-Cilag International NV) though Duomeisu^®^ was demonstrated to be bioequivalent to Caelyx^®^ [44]. Nowadays, it is clearly known that patients with HER2-positive MBC who did not receive anti-HER2 targeted therapy have the worst prognosis (10.1200/jco.2008.19.9844). Furthermore, PLD or PLD-based chemotherapy in combination with trastuzumab was also demonstrated to be safe and effective as a first-line treatment for HER2-positive MBC without an increased risk of cardiac toxicity[23, 28, 45].

More recently, eribulin, a novel non-taxane microtubule inhibitor, was approved as monotherapy for the treatment of MBC previously treated with anthracyclines and taxanes based on results of EMBRACE [46] and Study 301[47]. In the EMBRACE study, eribulin achieved median PFS of 3.7 months (95% CI 3.3–3.9) and OS of 13.1 months (95% CI 11.8–14.3) [46]. In Study 301, median PFS and OS for eribulin were 4.1 months (95% CI 3.5–4.3) and 15.9 months (95%: 15.2–17.6), respectively [47]. In Study 304, a phase III trial comparing eribulin to vinorelbine in Chinese patients with locally recurrent or MBC (2–5 prior chemotherapy regimens, including an anthracycline and a taxane), median PFS and OS with eribulin were 2.8 months and 13.4 months, respectively [48]. The results for PLD in our study were favorable to those for eribulin in EMBRACE and Study 304, probably attributable in part to the exclusion of patients with HER2-positive MBC in our study. Conversely, these results demonstrated that PLD could be comparable to eribulin for HER2-negative MBC heavily pretreated with conventional anthracycline and taxanes.

Currently, novel antibody–drug conjugates (ADCs) are rapidly evolving therapies for heavily pretreated HER2-negative MBC, including trastuzumab deruxtecan (T-DXd) [49] and sacituzumab govitecan (SG) [50, 51]. Interestingly, T-DXd is a novel anti-HER2-targeting ADC [52] that surprisingly improved both PFS and OS in patients with pretreated HER2-low MBC [49]. SG is a novel ADC consisting of anti-trophoblast cell surface antigen-2 (Trop-2) monoclonal antibody conjugated to an active metabolite of irinotecan[53]. Biomarker analyses in the ASCENT study found patients with high, medium, and low H-scores for Trop-2 expression had median PFS times of 6.9, 5.6, and 2.7 months, respectively [54]. Currently, chemotherapy remains one of the main therapies for MBC [55]. In our study, PLD showed a median PFS and OS of 4.0 and 16.3 months in the fourth/fifth-line subgroup, indicating that PLD is a reasonable treatment option, especially in developing countries considering the price and availability of ADCs. In the era of ADCs, further investigation is warranted to explore PLD as a payload for newer ADCs.

The overall safety profile was good in this study. No grade 4/5 AEs were observed. The most frequently reported AEs were leukopenia, fatigue, and neutropenia. The most common grade 3 AEs were neutropenia (7.3%) and fatigue (4.9%). All grades of PPE were 24.4% with 2.4% grade 3, and all grades of stomatitis 9.5% with no grade 3. Only three patients (7.3%) experienced grade 1 alopecia. Regarding cardiotoxicity, most patients (97.6%) had no significant decrease in LVEF during treatment, and only one patient experienced a decrease in LVEF from 70% at baseline to 62% after five cycles of PLD. These findings were consistent with those in the previous studies of PLD 10 mg/m^2^ every week [39, 43].

This study had several limitations. First, this study had a single-center, open-label design with no control arm. Second, the implementation of the study took longer than originally planned. The slow enrollment of patients was aggravated by the COVID-19 pandemic. Third, this was an exploratory trial with small sample sizes.

## Conclusion

In summary, our study demonstrated that PLD (Duomeisu^®^) monotherapy at 40 mg/m^2^ every 4 weeks was effective and safe in patients with HER2-negative MBC heavily pretreated with conventional anthracycline and taxanes, indicating that this treatment is a viable treatment option for this population.

## Electronic supplementary material

Below is the link to the electronic supplementary material.

Electronic supplementary material 1 (DOC 34 kb)

## Data Availability

The datasets used and/or analyzed during the current study are available from the corresponding author upon reasonable request.

## Abbreviations

ADC: Antibody–drug conjugate
AE: Adverse event
AIDS: Acquired immune deficiency syndrome
CBR: Clinical benefit rate
CI: Confidence interval
CR: Complete response
DCR: Disease control rate
HER2: Human epidermal growth factor receptor 2
HR: Hormone receptor
LVEF: Left ventricular ejection fraction
MBC: Metastatic breast cancer
ORR: Objective response rates
OS: Overall survival
PFS: Progression-free survival
PLD: Pegylated liposomal doxorubicin
PPE: Palmar-plantar erythrodysesthesia
PR: Partial response
RECIST: Response Evaluation Criteria for Solid Tumor
SD: Stable disease
SG: Sacituzumab govitecan
T-DXd: Trastuzumab deruxtecan
Trop-2: Trophoblast cell surface antigen-2
ULN: Upper limit of normal
