# Supplementary material for: Pegylated liposomal doxorubicin (Duomeisu®) monotherapy in patients with HER2-negative metastatic breast cancer heavily pretreated with anthracycline and taxanes: a single-arm, phase II study
Source: Breast Cancer Res Treat. 2023 Mar 6;199(1):67–79. doi: 10.1007/s10549-023-06894-3 (PMC9986665; doi:10.1007/s10549-023-06894-3)
Supplement: Supplementary file 1 — Electronic supplementary material 1 (DOC 34 kb) [file 10549_2023_6894_MOESM1_ESM.doc]

**Supplemental Methods**

**Dose modifications**

In the present study, adverse events and toxic reactions were evaluated according to the National Cancer Institute Common Terminology Criteria for Adverse Events (CTCAE, version 4.0). A PLD dose adjustment scheme was implemented because of toxicity. If LVEF decreased by ≥20% from baseline or by ≥10% from baseline to <50%, the PLD was discontinued, and the patient was withdrawn from the study. If a patient experienced ≥grade 2 other cardiotoxicity or PPE, PLD was resumed with a 25% dose reduction after the toxicity was resolved to grade 0-1. If a patient experienced grade 3 or 4 neutropenia, then prophylactic colony-stimulation factor could be administered 48 hours after completion of the next cycle of PLD without dose modification. For all other grade 3/4 hematologic or non-hematologic toxicities, PLD treatment was delayed until recovery to grade 0-1 and then the PLD dose was reduced by 25% in the subsequent cycles. A maximum of two dose adjustments was permitted per patient.
